# Supplementary material for: A Hierarchical Attractor Network Model of perceptual versus intentional decision updates
Source: Nat Commun. 2021 Apr 1;12:2020. doi: 10.1038/s41467-021-22017-2 (PMC8016916; doi:10.1038/s41467-021-22017-2)
Supplement: Supplementary file 1 — Supplementary Information [file 41467_2021_22017_MOESM1_ESM.pdf]

Supplementary Information

A Hierarchical Attractor Network Model of perceptual versus intentional decision updates

Löffler, Anne, Sylaidi, Anastasia, Fountas, Zafeirios, & Haggard, Patrick

Corresponding Author: Anne Löffler

Email address: al3928@columbia.edu

Supplementary Figures

Supplementary Figure 1

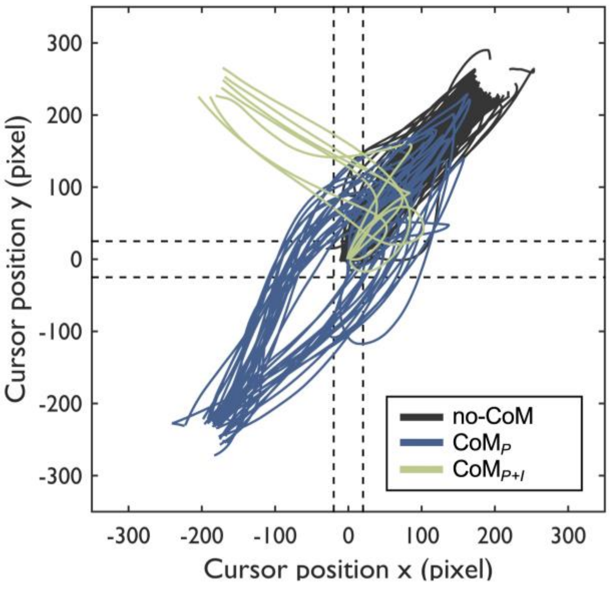

Supplementary Figure 1. Single-trial movement trajectories in test trials of one participant in Exp. 1. For illustration purposes, trajectories were mirrored such that the initial movement was always directed towards the upper right target, but ended in a different target depending on the class of movement trajectory (black = no Change of Mind; blue = 'perceptual Change of Mind'; green = 'perceptual + intentional Change of Mind'). Dashed lines indicate the boundary coordinates that were used as criteria for the classification of Changes of Mind.

Supplementary Figure 2

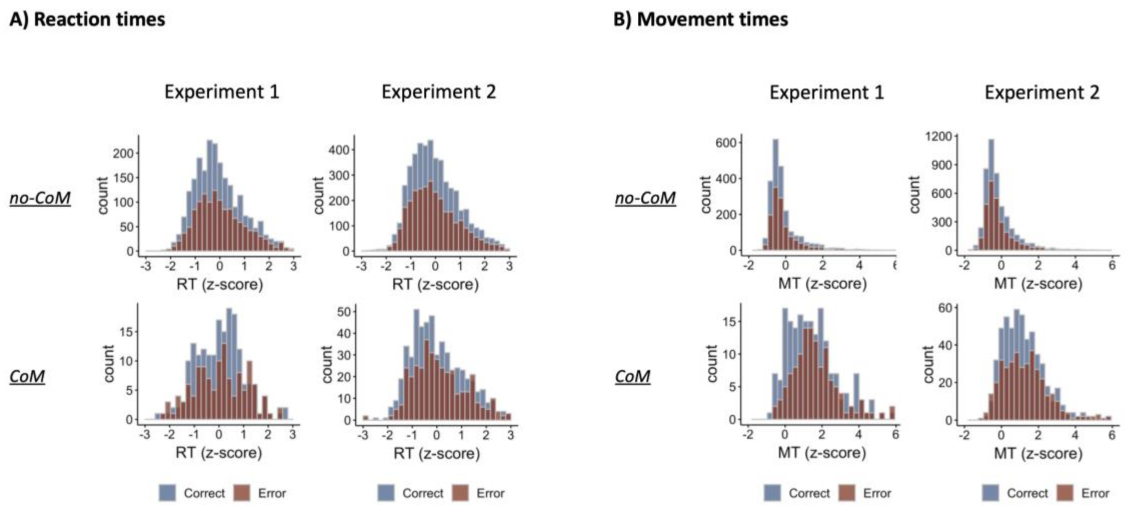

Supplementary Figure 2. Histograms of reaction times (A) and movement times (B) in test trials, separately for Exp. 1 and 2. For illustration purposes, reaction times (RTs) and movement times (MTs) were *z*-scored for each subject. Trials were then split into error (red) vs. correct trials (blue), and no-CoM (top) vs. CoM trials (bottom).

A linear mixed-effects regression with (non-standardized) RTs as outcome variable and Experiment (1 vs. 2), Error (correct vs. error), and CoM (no-CoM vs. CoM) as fixed effects revealed that RTs were significantly slower in error trials compared to correct trials (b = 7.76, 95% CI [0.78, 14.74], t(12344.8) = 2.18, p = .029). This was particularly pronounced in Exp. 2 (interaction Experiment x Error: b = -8.96, 95% CI [-17.35, -0.57], t(12342.6) = -2.09, p = .036). None of the other main effects or interactions were significant (all p > .05). Note that only correct trials were included in all the RT analyses reported in the main text, thus avoiding that differences in RTs between correct vs. error trials confounded RT results. For MTs, a significant effect of CoM was observed (b = 484.4, 95% CI [439.8, 528.9], t(12401.1) = 21.31, p < .001), reflecting the fact that CoM trials were associated with longer MTs than no-CoM trials. This effect was particularly pronounced in error trials (sign. interaction CoM x Error: b = 85.4, 95% CI [15.6, 155.2], t(12395.7) = 2.40, p = .017). The prolongation of MTs in error vs. correct CoM may have been caused by 1) erroneous CoMs occurring later during the movement than correct CoM, thus increasing overall path length, and/or 2) participants slowing down due to uncertainty in erroneous CoM compared to correct CoM. While we observed a modest trend for both potential mechanisms (results not shown), neither effect reached statistical significance (p > .05). Hence, the prolongation of MTs in error vs. correct CoMs may have resulted from a mixture of effects across different participants/trials. Finally, a main effect of Experiment on MTs was observed (b = 227.9, 95% CI [76.2, 379.5], t(33.4) = 3.03, p = .005), which was expected given that movement paths were longer in Exp. 2 than Exp. 1 by design (see *Methods* section). As expected, this difference was particularly pronounced in CoM trials (sign. interaction between Experiment x CoM: b = 103.7, 95% CI [51.7, 155.6], t(12400.3) = 3.91, p < .001).

Supplementary Figure 3

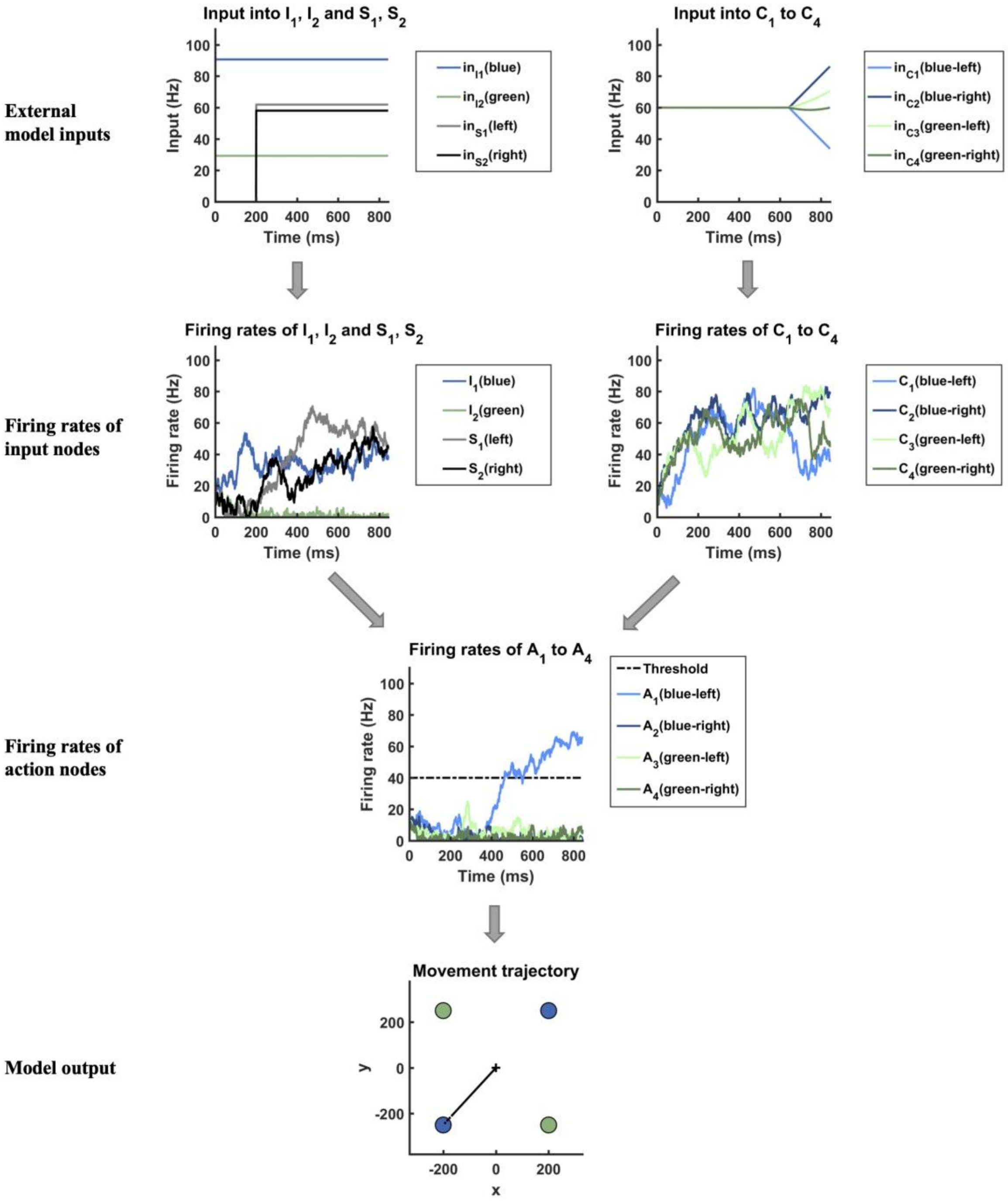

Supplementary Figure 3. Single-trial simulation of Hierarchical Attractor Network Model: Example of trial without Change of Mind. External model inputs (top row) are applied to intention, sensory and cost nodes (second row). Information is then combined by action nodes (third row) that determine the resulting movement trajectory (bottom row). In this example, the model correctly selects the left-blue target. After threshold crossing, the corresponding movement is initiated with a motor delay of 180 ms, and firing rates continue to be updated for a total non-decision time of 380 ms. Furthermore, inputs into cost nodes change after action initiation, according to the distance of the current cursor position to each target. Note that updates in cost nodes lag behind updates in action nodes due to the motor delay (i.e., costs only start changing 180 ms after a given action node has crossed the threshold).

Supplementary Figure 4

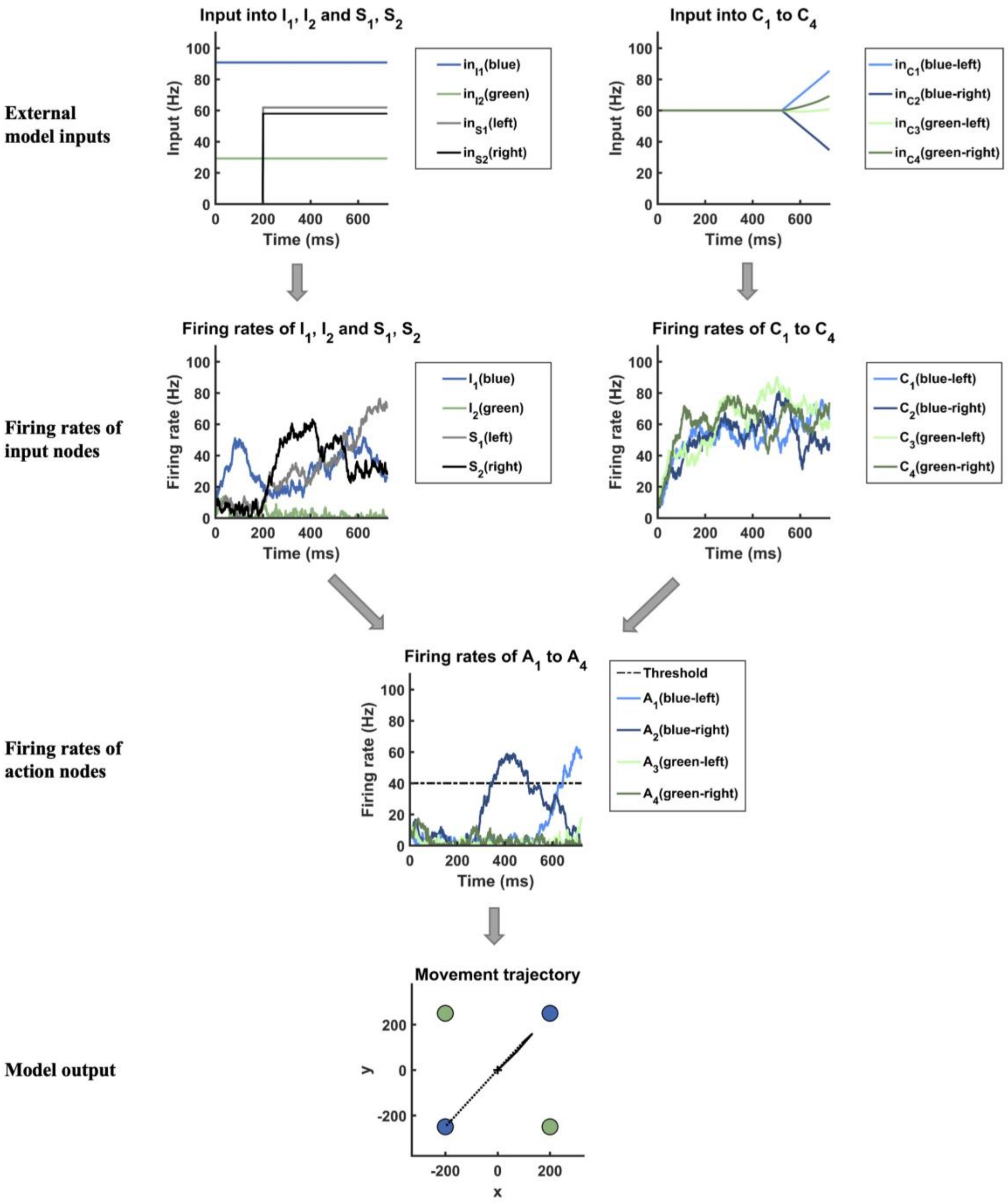

Supplementary Figure 4. Single-trial simulation of Hierarchical Attractor Network Model: Example of trial with perceptual Change of Mind (CoM$_P$).

Supplementary Figure 5

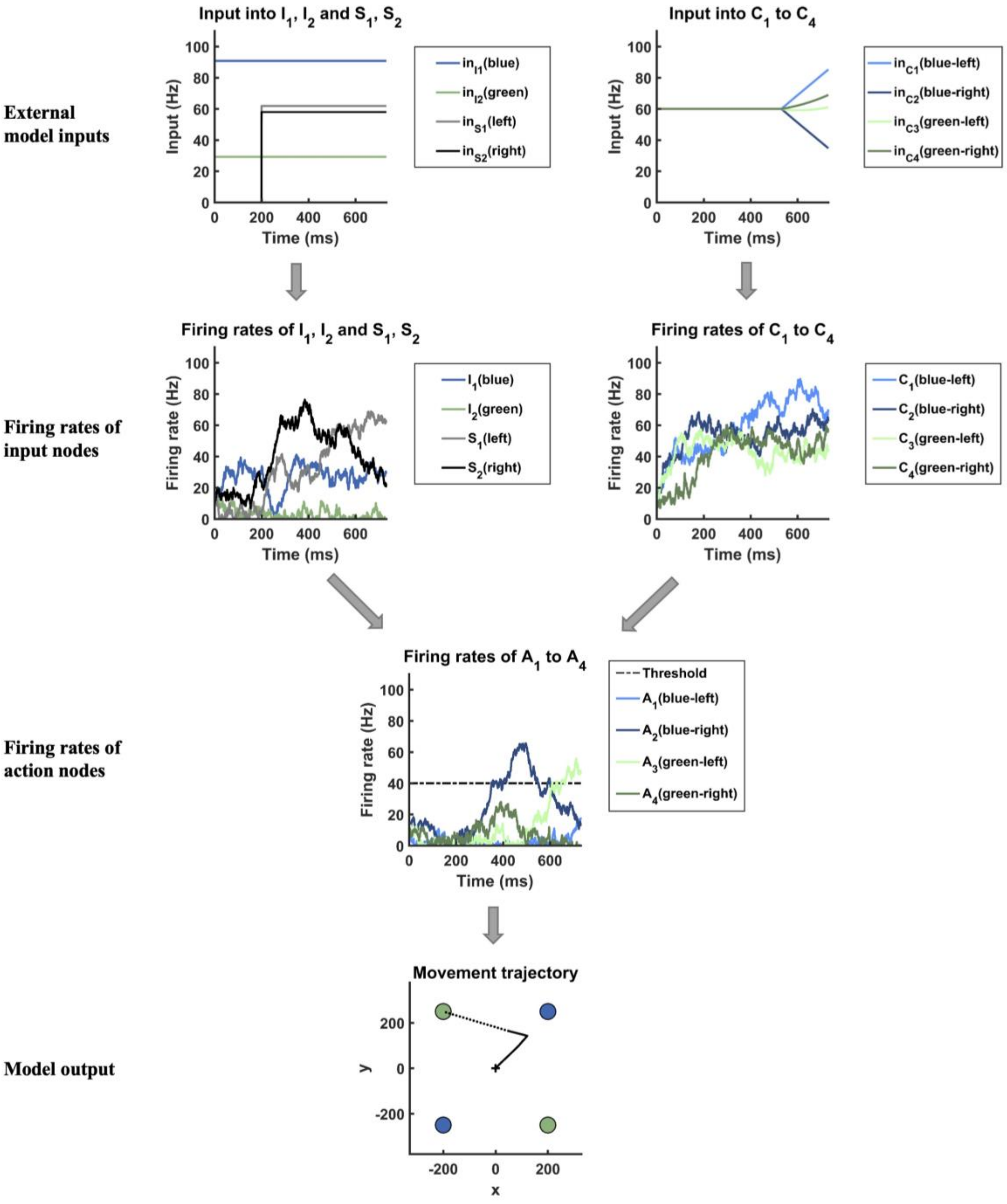

Supplementary Figure 5. Single-trial simulation of Hierarchical Attractor Network Model: Example of trial with perceptual + intentional Change of Mind (CoM_{P+I}).

Supplementary Figure 6

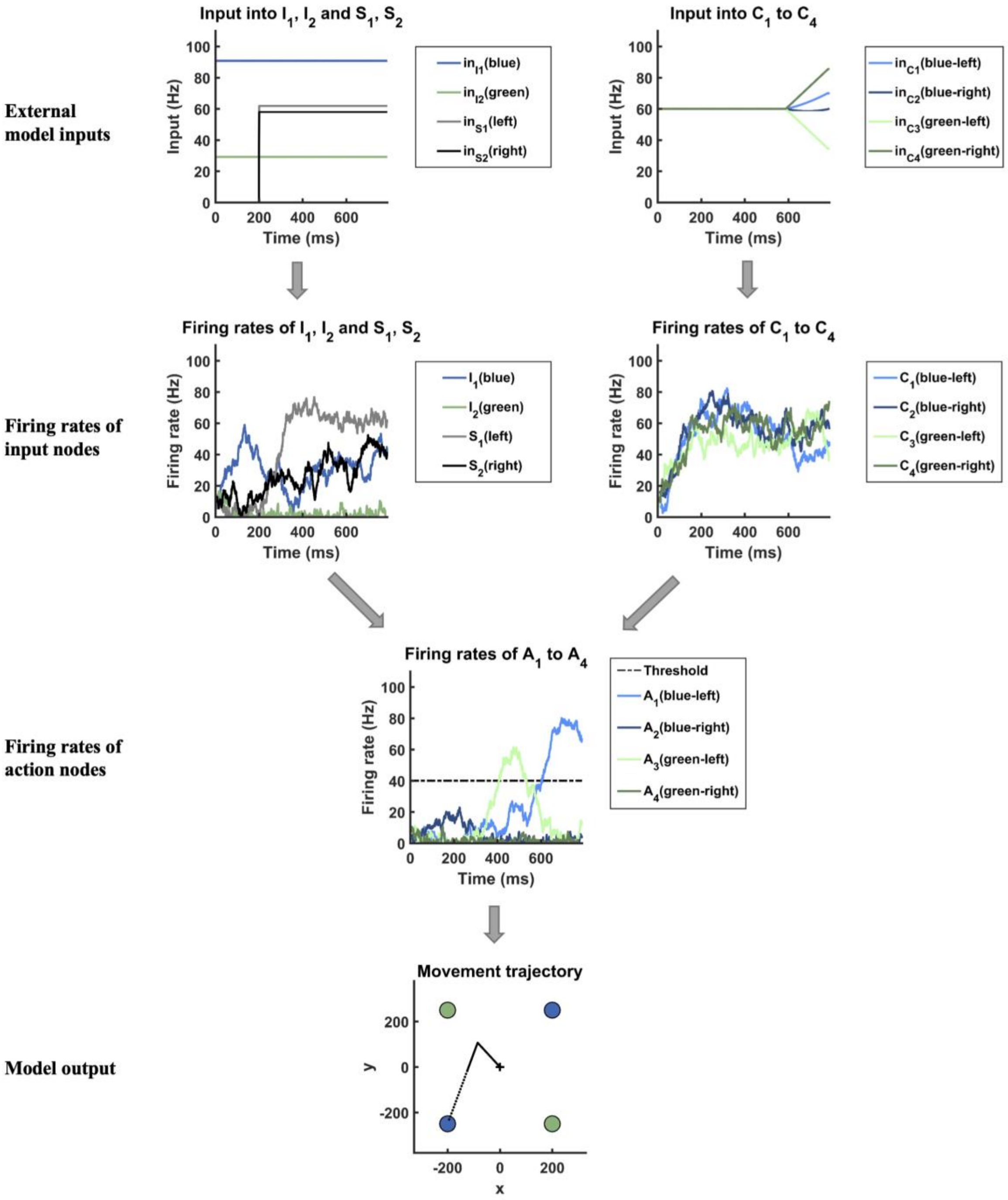

Supplementary Figure 6. Single-trial simulation of Hierarchical Attractor Network Model: Example of trial with vertical Change of Mind.

Supplementary Figure 7

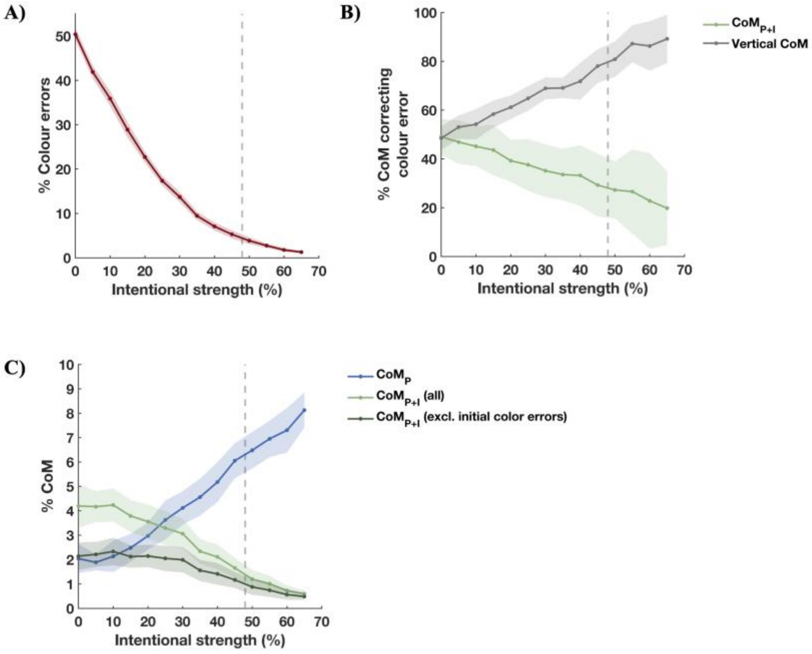

Supplementary Figure 7. Effect of intentional strength on Change of Mind (CoM) in Hierarchical Attractor Network Model. A) Stronger colour intentions reduce % initial colour errors. B) Stronger colour intentions result in more colour errors being corrected with vertical movement corrections (grey) as opposed to CoM$_{P+I}$ (green). C) Stronger colour intentions increase CoM$_{P}$ (blue) but reduce % CoM$_{P+I}$ (green), even when trials with initial colour errors are excluded (dark green). [M ± 1 SD]. In A)–C) each data point represents the mean (± 1 SD) of n = 30 model simulations with 1000 trials each.

Simulations with different degrees of intentional strength were performed (*col* = 0–65%), while all other model parameters were kept constant. In line with our behavioural findings, stronger colour intentions in the model predicted a decrease in the relative frequency of CoM$_{P+I}$ out of all CoM (**Fig. 6C** in main text). Note that stronger colour intentions also caused fewer initial colour errors (**Supplementary Figure 7A**). However, even when trials with colour errors were excluded, CoM$_{P+I}$ decreased relative to CoM$_{P}$ (**Supplementary Figure 7C**). This was due to the fact that stronger colour intentions shifted the percentage of colour errors that were corrected disproportionally towards vertical movement corrections (between targets of different colour on the *same* side of the screen), whereas CoM$_{P+I}$ that corrected initial colour errors were less frequent for stronger intentions (**Supplementary Figure 7B**). Hence, the effect of intentional strength on CoM$_{P+I}$ was not mediated by differences in initial colour errors.

Supplementary Figure 8

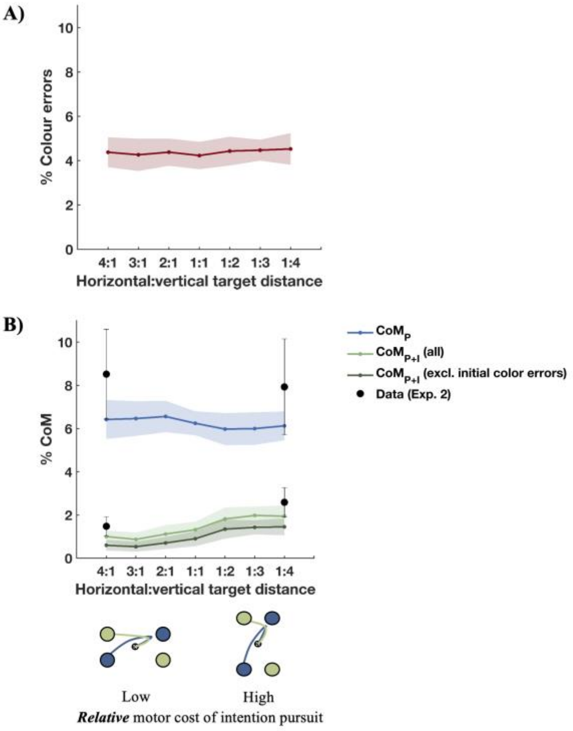

Supplementary Figure 8. Effect of target distance (i.e., motor costs) on CoM in Hierarchical Attractor Network Model. A) Relative target distance does not affect % colour errors. B) Closer horizontal targets increase % CoM_{P+I} (green), even when trials with initial colour errors were excluded (dark green). In A) and B), each data point represents the mean (± 1 SD) of n = 30 model simulations with 1000 trials each. Black data points in B) represent behavioural results from Exp. 2 (n = 16; M ± 1 SEM).

The effect of target distance was simulated by changing the ratio of x:y target coordinates, while keeping all other model parameters constant. For example, in Exp. 2, target distances from the centre were x = 300 and y = 100, indicating a ratio of 3:1, and thus a far horizontal distance that causes low *relative* costs of intention pursuit. By contrast, a ratio of 1:3 (x = 100 and y = 300) indicates close horizontal targets, and thus a high relative cost of intention pursuit that should increase the frequency of intention reversals. Simulations with ratios ranging from 4:1 to 1:4 were performed. Crucially, the overall distance from the centre was equal for all targets, and hence, initial costs of each action were constant across all simulations. However, the relative change in costs after action onset varied as a function of x:y ratios in target locations. In line with our behavioural results, the model predicted an increase in CoM_{P+I} with closer horizontal targets (**Fig. 6E** in main text). Importantly, target distance did not affect the rate of colour errors (**Supplementary Figure 8A)**, and hence, excluding trials with initial colour errors yielded the same pattern of results (**Supplementary Figure 8B**). Thus, differences in changes of intention across the different target distance conditions were not driven by potential differences in correcting initial colour errors.

Supplementary Tables

Supplementary Table 1. Optimized parameters in Hierarchical Attractor Network Model.

**Model parameters**	**Initial value**	**Fine-tuned value**
Connectivity weights w		
w intention → action	1.00	0.97
w sensory → action	1.50	1.50
w cost → action	-1.00	-0.97
w sensory → sensory	0.25	0.25
w lateral inhibition	-0.50	-0.52
Decision evidence		
sensory evidence (% *coh*)	3.20%	1.03%
intentional strength (% *col*)	51.2%	48.0%
Hierarchical noise control		
hierarchical control (*h*)	1.00	2.01

Supplementary Table 2. Model predictions and participants' behaviour in Exp. 1 [*M* (*SD*)].

**Outcome variable**	**Model prediction**	**Behaviour (Exp. 1)**
Reaction times (ms)	645.7 (4.9)	570.5 (58.3)
% CoM_P	6.33 (0.8)	5.93 (5.5)
% CoM_{P+I}	1.41 (0.4)	1.71 (2.2)
% Vertical colour change	3.58 (0.6)	3.24 (2.6)
% double CoM	1.61 (0.4)	0.83 (1.0)
% perceptual choice accuracy	54.5 (1.5)	56.6 (9.1)
% colour choice accuracy	95.6 (0.6)	–
% misses (RT > 1000 ms)	8.6 (0.9)	9.3 (6.5)
% early responses (before stimulus onset)	0.4 (0.2)	–

Supplementary Notes

Supplementary Note 1: Task performance in Experiment 2

As in Exp. 1, accuracy of the perceptual choice was significantly lower in test trials (*M* = 58.5%, *SD* = 5.2%) compared to easy trials (*M* = 96.2%, *SD* = 3.4%, $t(15) = 26.54$, $p < .001$, d = 6.64), and perceptual CoM occurred more frequently in test (*M* = 10.3%, *SD* = 9.9%) than easy trials (*M* = 3.3%, *SD* = 4.1%, $t(15) = 3.15$, $p = .007$, $d = 0.79$). Additionally, similarly to Exp. 1, the majority of perceptual CoM in Exp. 2 corrected an initial error (*M* = 61.5%, *SD* = 10.8%, $t(15) = 4.26$, $p < .001$, $d = 1.07$). A mixed-effects logistic regression model with accuracy as outcome variable and CoM type (CoM_P/CoM_{P+I}) and Exp (1 vs. 2) did not reveal any significant main effects or interactions (all $p > .05$), indicating that across both experiments, accuracy was comparable for different types of CoM.

Furthermore, in trials where participants did not have to verbalise their colour choice, accuracy showed a trend towards being lower in conflict (*M* = 93.1%, *SD* = 5.7%) than easy trials (*M* = 95.7%, *SD* = 3.5%; $t(15) = 1.89$, $p = .078$, $d = 0.47$). In contrast to Exp. 1, no difference in RTs was observed between conflict trials (*M* = 560.8 ms, *SD* = 47.5 ms) and easy trials (*M* = 563.0 ms, *SD* = 48.5 ms, $t(15) = 0.38$, $p = .712$, $d = 0.09$). However, the rate of misses (i.e., trials in which movement initiation exceeded the response deadline) was numerically increased in conflict trials (*M* = 3.3%, *SD* = 3.3%; easy trials: *M* = 2.0%, *SD* = 3.1%; $t(15) = 1.29$, $p = .216$, $d = 0.32$). Additionally, there were significantly more corrective movements in conflict trials (*M* = 5.5%, *SD* = 6.3%) than there were CoM in easy trials (*M* = 3.4%, *SD* = 5.5%, $t(15) = 3.51$, $p = .003$, $d = 0.88$) suggesting that, as in Exp. 1, participants generated initial colour intentions that resulted in response costs when external information did not match the endogenous intention.

Supplementary Note 2: Model simulations of conflict trials

To simulate the effect of intentional strength on RT costs in conflict trials (**Fig. 6D** in main text and **Supplementary Figure 7**), conflict and easy trials were simulated using the Hierarchical Attractor Network Model. For conflict trials, the model was changed such that each intention node

mapped onto the two targets on the same (left/right) side (i.e., blue → both left actions A_1 and A_3; green → both right actions A_2 and A_4). The true colour intention of a given trial was selected randomly to induce a mismatch between colour intention and perceptual input on ~50% of trials (e.g., intention = green and both green targets are on the right side, but dot-motion direction = left). Additionally, in line with the behavioural task, both conflict and easy trials were simulated with a high motion coherence level (*coh* = 50%) to ensure that differences in RTs are not driven by perceptual difficulty. Note however that the precise level of motion coherence is of little importance for this simulation since RT costs are relative, i.e., they reflect the difference in RTs between conflict and easy trials (for correct perceptual decisions). As expected, the model predicted that stronger colour intentions result in higher RT costs in conflict trials (**Fig. 6D** in main text).
