# Supplementary material for: A Hierarchical Attractor Network Model of perceptual versus intentional decision updates
Source: Nat Commun. 2021 Apr 1;12:2020. doi: 10.1038/s41467-021-22017-2 (PMC8016916; doi:10.1038/s41467-021-22017-2)
Supplement: Supplementary file 3 — Reporting Summary [file 41467_2021_22017_MOESM3_ESM.pdf]

Corresponding author(s): Anne Loffler

Last updated by author(s): Nov 13, 2020

Reporting Summary

Nature Research wishes to improve the reproducibility of the work that we publish. This form provides structure for consistency and transparency in reporting. For further information on Nature Research policies, see our Editorial Policies and the Editorial Policy Checklist.

Statistics

For all statistical analyses, confirm that the following items are present in the figure legend, table legend, main text, or Methods section.

n/a	Confirmed	
☐	☒	The exact sample size (*n*) for each experimental group/condition, given as a discrete number and unit of measurement
☐	☒	A statement on whether measurements were taken from distinct samples or whether the same sample was measured repeatedly
☐	☒	The statistical test(s) used AND whether they are one- or two-sided<br>*Only common tests should be described solely by name; describe more complex techniques in the Methods section.*
☐	☒	A description of all covariates tested
☐	☒	A description of any assumptions or corrections, such as tests of normality and adjustment for multiple comparisons
☐	☒	A full description of the statistical parameters including central tendency (e.g. means) or other basic estimates (e.g. regression coefficient) AND variation (e.g. standard deviation) or associated estimates of uncertainty (e.g. confidence intervals)
☐	☒	For null hypothesis testing, the test statistic (e.g. *F*, *t*, *r*) with confidence intervals, effect sizes, degrees of freedom and *P* value noted<br>*Give P values as exact values whenever suitable.*
☒	☐	For Bayesian analysis, information on the choice of priors and Markov chain Monte Carlo settings
☒	☐	For hierarchical and complex designs, identification of the appropriate level for tests and full reporting of outcomes
☐	☒	Estimates of effect sizes (e.g. Cohen's *d*, Pearson's *r*), indicating how they were calculated

Our web collection on statistics for biologists contains articles on many of the points above.

Software and code

Policy information about availability of computer code

Data collection	All data were collected using custom code written in Matlab R2014a and the Psychophysics Toolbox. Motion stimuli were generated using the Variable Coherence Random-Dot Motion code (https://shadlenlab.columbia.edu/resources/VCRDM.html).
Data analysis	The main analyses using linear mixed-effects models were performed with the lme4 package in R version 3.2.4. Movement trajectory analyses were conducted in Matlab R2014b. All other analyses (comparison of means with ANOVAs/t tests) were performed in IBM SPSS Statistics for Windows, version 21. Model fitting was performed using Python 3.7.

For manuscripts utilizing custom algorithms or software that are central to the research but not yet described in published literature, software must be made available to editors and reviewers. We strongly encourage code deposition in a community repository (e.g. GitHub). See the Nature Research guidelines for submitting code & software for further information.

Data

Policy information about availability of data

All manuscripts must include a data availability statement. This statement should provide the following information, where applicable:

- Accession codes, unique identifiers, or web links for publicly available datasets
- A list of figures that have associated raw data
- A description of any restrictions on data availability

The datasets generated during and/or analyzed during the current study are publicly available on the Open Science Framework, https://osf.io/wbex8/ (doi:10.17605/OSF.IO/WBEX8).

Field-specific reporting

Please select the one below that is the best fit for your research. If you are not sure, read the appropriate sections before making your selection.

☒ Life sciences ☐ Behavioural & social sciences ☐ Ecological, evolutionary & environmental sciences

For a reference copy of the document with all sections, see nature.com/documents/nr-reporting-summary-flat.pdf

Life sciences study design

All studies must disclose on these points even when the disclosure is negative.

Sample size	Based on pilot data (N = 13), a power calculation was conducted to determine the sample size required to obtain a percentage of CoM that is significantly greater than zero across participants. With an observed effect size of d = 0.67, α = .05, and β = .8, the analysis resulted in N = 16. In anticipation of attrition, 21 participants were recruited for each experiment. Based on performance criteria (see below), some participants were excluded from all analysis, resulting in a final sample size of N = 16 in Exp. 1 and N = 17 in Exp. 2.
Data exclusions	In Exp. 1, one participant did not reach the performance criterion in the training session (70% accuracy in trials with 35% motion coherence) and another participant withdrew after training. Two further participants were excluded, one due to technical issues during data collection and one due to strategic decision delay in the task (frequent stopping after response initiation, see Methods section for details). In Exp. 2, 3 participants did not reach the performance criterion during training and two further participants were excluded due to poor performance in the test session (> 15% errors or misses in easy trials). All exclusion criteria were established prior to the study based on a pilot experiment.
Replication	Each experiment was only conducted once and was not replicated independently. However, the overall behavioural results in Exp. 2 were consistent with the main findings from Exp. 1 (e.g., overall rate of CoM; see Supplementary Material S2), despite a slightly different task design.
Randomization	Participants were not allocated into different experimental groups. Instead, most experimental variables of interest (e.g., easy/test/conflict trial condition, close/far target distance and early/late onset delay) were manipulated within participants. Trial conditions were presented in a pseudo-random order ensuring that all conditions were presented in a counterbalanced manner. Intentional strength varied between participants, however, this variable was not manipulated experimentally, and hence, no randomization was possible.
Blinding	All experimental conditions were within-subject manipulations of randomly assigned trial conditions, and thus, blinding with regard to group allocations was not required for our study.

Reporting for specific materials, systems and methods

We require information from authors about some types of materials, experimental systems and methods used in many studies. Here, indicate whether each material, system or method listed is relevant to your study. If you are not sure if a list item applies to your research, read the appropriate section before selecting a response.

Materials & experimental systems

n/a	Involved in the study	
☒	☐	Antibodies
☒	☐	Eukaryotic cell lines
☒	☐	Palaeontology and archaeology
☒	☐	Animals and other organisms
☐	☒	Human research participants
☒	☐	Clinical data
☒	☐	Dual use research of concern

Methods

n/a	Involved in the study	
☒	☐	ChIP-seq
☒	☐	Flow cytometry
☒	☐	MRI-based neuroimaging

Human research participants

Policy information about studies involving human research participants

Population characteristics	For Exp. 1, twenty-one right-handed participants were recruited. After exclusion (see above), the final sample consisted of 17 participants (13 female, age: M = 22.6 yr, SD = 3.1). For Exp. 2, twenty-one right-handed participants were initially invited for the experiment. After exclusion (see above), a final sample of 16 participants (11 female, age: M = 23.2 yr, SD = 2.9) was included in the analyses.
Recruitment	All participants were recruited through the ICN (Institute of Cognitive Neuroscience at UCL) subject database. Invitations were sent out to a random sample of the pool for each experiment. Any self-selection bias or other biases are unlikely to have affected the results of the study given that all subjects were naive with respect to the specific aim and hypotheses of our experiments.

Ethics oversight	The study was approved by the UCL Research Ethics Committee and all participants provided written informed consent prior to the study.

Note that full information on the approval of the study protocol must also be provided in the manuscript.
